# Resolution of Erythema Nodosum Following FLT3-Targeted Therapy in Acute Myeloid Leukemia: A Case Report

**DOI:** 10.7759/cureus.78872

**Published:** 2025-02-11

**Authors:** Risa Nakane, Romane Teshima, Natsuko Saito-Sasaki, Yu Sawada

**Affiliations:** 1 Dermatology, University of Occupational and Environmental Health, Kitakyushu, JPN

**Keywords:** case report, erythema nodosum, flt3-itd mutation, leukemia, paraneoplastic syndrome

## Abstract

A 59-year-old woman with persistent lower-leg erythema and pain, initially misdiagnosed as cellulitis, was later diagnosed with erythema nodosum (EN) and acute myeloid leukemia (AML), French-American-British (FAB) subtype M2, carrying a FLT3-internal tandem duplication (ITD) mutation. FLT3 mutations, common in AML-M2 and M4, activate PI3K/AKT and STAT5 pathways, driving cytokine production (tumor necrosis factor-alpha (TNF-α), IL-1β, IL-6, and granulocyte-macrophage colony-stimulating factor (GM-CSF)) and contributing to EN as a paraneoplastic phenomenon. This case highlights the role of FLT3 mutations in AML-associated inflammation and the importance of distinguishing paraneoplastic from drug-induced EN to guide treatment strategies.

## Introduction

Erythema nodosum (EN) is a panniculitis characterized by painful erythematous nodules and is associated with autoimmune diseases and malignancies [1-3], including acute myeloid leukemia (AML) [4]. In particular, FLT3 mutations, frequently found in AML-M2, drive abnormal cytokine production, which promote immune cell infiltration and inflammation [5]. These cytokines, also implicated in EN [6], suggest overlapping inflammatory mechanisms between AML with FLT3 mutations and EN. In this case, EN presented as the initial symptom of AML-M2 with FLT3 mutation and resolved following FLT3-targeted therapy, highlighting the therapeutic role of targeted treatment.

## Case presentation

**Figure 1:**
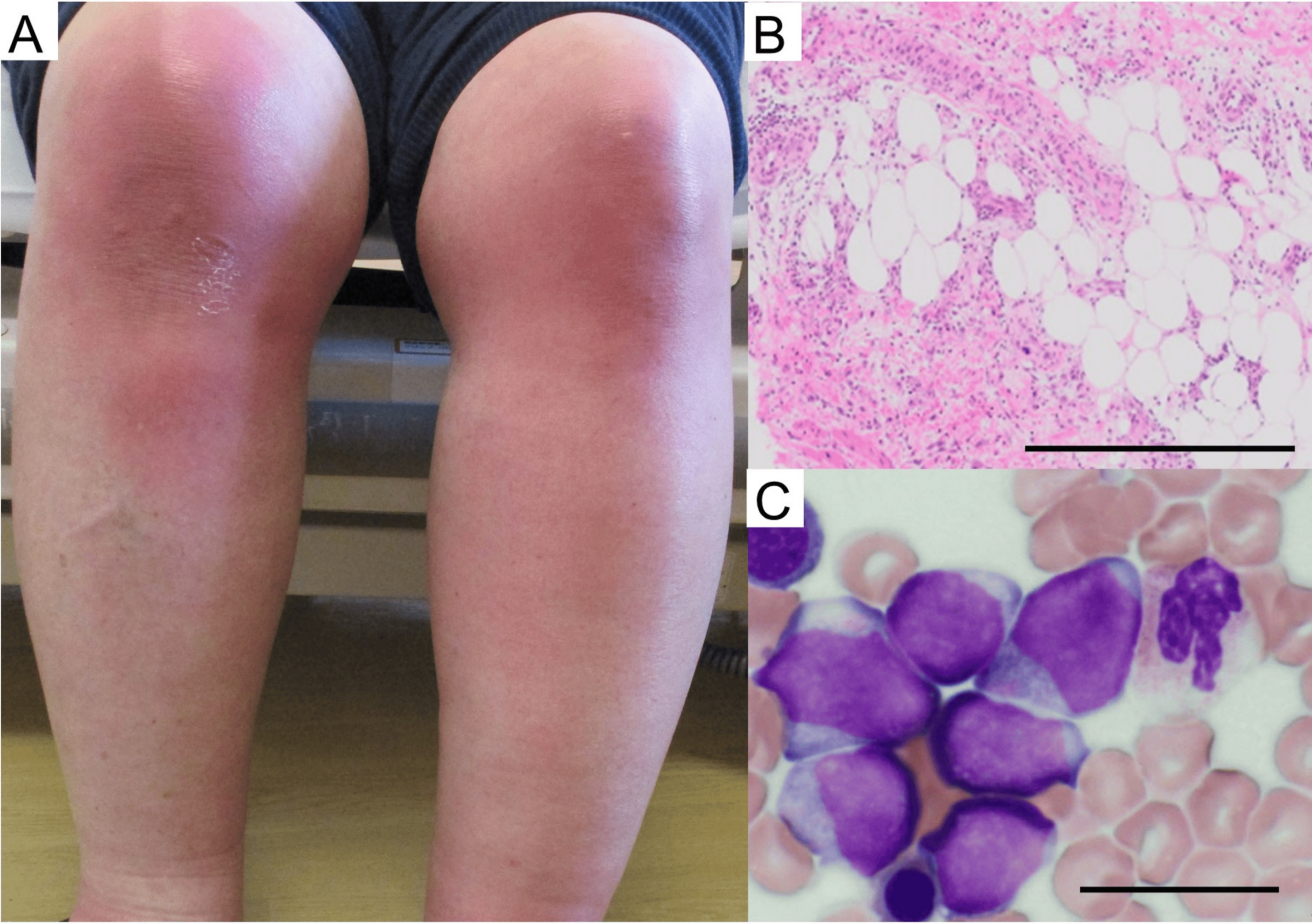
Clinical and pathological findings of erythema nodosum (EN) associated with AML-M2. (A) Multiple erythematous, tender nodules on the lower legs, characteristic of EN. (B) Skin biopsy showing septal panniculitis without vasculitis, consistent with EN (hematoxylin and eosin staining, scale bar = 500 μm). (C) Bone marrow biopsy showing hypercellularity with increased blasts, indicative of AML-M2 (hematoxylin and eosin staining, scale bar = 20 μm). AML: acute myeloid leukemia.

A 59-year-old woman, working part-time at a supermarket, developed swelling, erythema, and pain in both knees and her left ankle after a fall. The lesions initially appeared as erythematous, tender nodules on both lower legs and progressively increased in number and size over the first 10 days. She was suspected of having cellulitis and remained unresponsive to antibiotics for one week, leading to a referral for further evaluation. Physical examination revealed multiple tender, erythematous nodules on both lower legs, without signs of ulceration or drainage (Figure 1A). The differential diagnoses included cellulitis, sarcoidosis, erythema induratum, and lupus panniculitis. To confirm the diagnosis of her skin eruption, a skin biopsy of the affected area revealed septal panniculitis without vasculitis, consistent with EN (Figure 1B). The laboratory findings reveal notable abnormalities, including an elevated white blood cell count (10.3 × 10³/μl), thrombocytosis (542 × 10³/μl), elevated lactate dehydrogenase (LDH) (179 U/l), and significantly increased C-reactive protein (CRP) (24.37 mg/dl). Most importantly, the presence of circulating blasts (2%) strongly suggests a potential hematologic malignancy, such as leukemia. This finding, combined with the refractory nature of EN and other hematologic abnormalities, justified the need for a bone marrow biopsy to confirm or rule out acute leukemia as the underlying cause. Given the refractory nature of her symptoms and abnormal hematologic findings, a bone marrow biopsy was performed (Figure 1C). The biopsy confirmed AML, French-American-British (FAB) subtype M2, with flow cytometry identifying a FLT3-internal tandem duplication (ITD) mutation.

Induction chemotherapy with idarubicin and cytarabine was initiated. On day 23, gilteritinib, a FLT3 inhibitor, was added to her regimen. The EN lesions began to resolve within a few days of chemotherapy initiation. When gilteritinib therapy was introduced, her erythematous nodules had completely disappeared, and inflammatory markers normalized. The patient remained in remission, with no recurrence of EN or AML during subsequent follow-up visits for one year.

## Discussion

EN is a rare manifestation of AML, with only two cases specifying the AML subtype: one with AML-M2 and another with AML-M4. In both cases, EN resolved with chemotherapy and recurred upon relapse, suggesting that EN may reflect disease activity. Certain clinical and laboratory findings in cases of EN can raise suspicion of an underlying malignancy, particularly when the condition is refractory to standard treatments. In this case, the combination of refractory EN, abnormal hematologic findings such as circulating blasts, thrombocytosis, and elevated LDH levels served as key indicators prompting further investigation into a potential hematologic malignancy, ultimately leading to the diagnosis of AML-M2 with a FLT3-ITD mutation.

Although Sweet syndrome, and less frequently pyoderma gangrenosum, are associated with AML [7,8], FLT3 mutations, including internal tandem duplication (ITD) and tyrosine kinase domain (TKD) mutations, are frequently observed in AML-M2 and AML-M4 [9]. This prevalence may result from the role of FLT3 mutations in promoting leukemic cell proliferation and survival through activation of the PI3K/AKT and STAT5 pathways [5]. These mutations drive aberrant cytokine production, including tumor necrosis factor-alpha (TNF-α), IL-1β, IL-6, and granulocyte-macrophage colony-stimulating factor (GM-CSF), contributing to the development of EN [6,10]. The connection between FLT3-driven inflammation and EN highlights how oncogenic mutations can trigger immune dysregulation, leading to paraneoplastic manifestations.

This case is the first to report EN in AML-M2 with a confirmed FLT3-ITD mutation, suggesting that FLT3 mutations may have a specific role in driving inflammatory processes linked to EN. The frequent association of FLT3 mutations with AML-M2 and AML-M4 subtypes could explain why EN appears in these subtypes [11,12]. The enhanced proliferative and inflammatory response in these subtypes may create a cytokine environment conducive to paraneoplastic phenomena like EN. The rapid resolution of EN with induction chemotherapy targeting FLT3 inhibition, in this case, strongly supports a paraneoplastic mechanism. These findings indicate that immune cells activated through STAT5 may contribute to its pathogenesis.

## Conclusions

This case demonstrates EN as a rare paraneoplastic manifestation of AML-M2 with a FLT3-ITD mutation. The rapid resolution of EN after FLT3-targeted therapy highlights its role as a potential indicator of disease activity. Clinicians should consider AML in patients with refractory EN to enable early diagnosis and treatment.
